# Influence of Training Load on Muscle Contractile Properties in Semi-Professional Female Soccer Players Across a Competitive Microcycle: A Pilot Study

**DOI:** 10.3390/s24216996

**Published:** 2024-10-30

**Authors:** Ezequiel Rey, María Lois-Abal, Alexis Padrón-Cabo, Miguel Lorenzo-Martínez, Pablo B. Costa

**Affiliations:** 1 Faculty of Education and Sport Sciences, University of Vigo, 36005 Pontevedra, Spain; marialoisabal@gmail.com (M.L.-A.); apadron@uvigo.gal (A.P.-C.); miguel.lorenzo.martinez@uvigo.gal (M.L.-M.); 2 Human Performance Laboratory, Center for Sport Performance, Department of Kinesiology, California State University, Fullerton, CA 92831, USA; pcosta@fullerton.edu

**Keywords:** MyotonPRO, football, stiffness, elasticity, muscle tone

## Abstract

This study aimed to evaluate changes in muscle contractile properties during a training microcycle in semi-professional female football players and explore their relationship with training load variables. Nineteen players (age: 23.9 ± 3.9 years; body mass: 60.6 ± 6.9 kg; height: 164.5 ± 6.7 cm) underwent myotonometric assessments of the biceps femoris (BF) and rectus femoris (RF) before and after the following training sessions: MD1 (i.e., 1 day after the match), MD3, MD4, and MD5. Training loads were quantified for each session, revealing significant variations, with MD4 exhibiting the highest values for high-speed running distance, number of sprints, and accelerations. Notably, MD3 showed the highest perceived exertion (RPE), while MD5 recorded the lowest total distance run. Myotonometric assessments indicated significant differences in stiffness of the RF in MD3 and BF in MD5, as well as RF tone in MD5. The findings underscore a notable relationship between training load and myotometric variables, particularly in muscle stiffness and tone. These results emphasize the need for further research to clarify how training loads affect muscle properties in female athletes.

## 1. Introduction

Women’s soccer is characterized by a unique and intermittent playing style, which involves numerous short, high-intensity actions interspersed with periods of lower intensity. During competitive matches, players typically cover an average total distance (TD) ranging from 9.2 to 11.3 km, with high-speed running distance (HSRD) varying between 1.2 and 2.7 km. The physiological demands of the game are reflected in players’ average heart rates (HR), which fluctuate between 152 and 186 beats per minute (bpm), corresponding to approximately 80% to 90% of their maximum heart rate (HRmax) [1].

Given the complexity of performance in team sports, it is crucial to implement daily variations in training load throughout a microcycle to maintain or enhance performance during the competitive season. Physical trainers play a pivotal role in this process by closely monitoring the impact of training sessions on players’ performance [2]. Consequently, tracking both training and match load in female soccer players has become an essential responsibility for physical trainers and sports scientists. This systematic monitoring enables technical staff to tailor training stimuli, manage recovery strategies, and mitigate fatigue and injury risk.

Training load can be categorized into two primary dimensions: external and internal [3]. External load refers to the mechanical intensity imposed on players through various exercises or tasks [4], while internal load encompasses the psycho-physiological responses elicited by the external load [3]. Although these two dimensions do not always exhibit a direct correlation, research has indicated that they can be interrelated depending on the specific variables analyzed [5]. To quantify external load in soccer, global positioning systems (GPS), local positioning systems, and inertial measurement units (IMUs) are commonly employed [6]. These devices provide valuable data on distances covered at various speed thresholds, changes in velocity (including accelerations, decelerations, and direction changes), and overall external intensity derived from IMUs.

Conversely, internal load is typically assessed using heart rate monitors and perceived exertion scales. Heart rate monitors capture metrics such as maximum heart rate (HRmax) and heart rate variability, while perceived exertion is often quantified using the Rate of Perceived Exertion (RPE) scale [7]. Internal load variables can be classified as either objective or subjective. Heart rate measurements provide insights into the aerobic demands of exercise, as there exists a strong relationship between oxygen consumption and heart rate variables, including HRmax and heart rate reserve [8,9]. However, heart rate is less reliable for capturing high-intensity anaerobic efforts, such as sprints and accelerations, which are prevalent in soccer [10]. Consequently, heart rate is more suitable for monitoring continuous efforts that remain below the anaerobic threshold [7].

The RPE scale, widely utilized due to its ease of application, holds particular relevance in women’s soccer, where budget constraints often limit access to advanced load monitoring equipment. RPE provides a cost-effective method that correlates well with heart rate-based metrics such as Training Impulse (TRIMP) [11]. Nevertheless, the reliability of RPE is contingent upon standardized instructions and the familiarity of players and coaches with the scale [12]. In male soccer, studies have demonstrated moderate to strong correlations between internal load measures, such as TRIMP and session-RPE (sRPE), and external load metrics, including TD covered, HSRD, and player load [13]. Conversely, the relationship between acceleration variables and RPE is moderate, indicating variability in the subjective perception of effort among players [14,15].

Despite the availability of methods for monitoring internal and external loads, there is a growing interest in assessing neuromuscular function to quantify training load. Techniques such as countermovement jumps (CMJ) are commonly employed in team sports; however, they have faced criticism for their lack of sensitivity in detecting changes in load [16]. Recent research has suggested that muscle stiffness, defined as the tension within the muscle–tendon unit, may serve as a more sensitive indicator of neuromuscular function and training load [17]. Elevated stiffness levels have been associated with both enhanced athletic performance and an increased risk of injury, indicating that an optimal range of stiffness is beneficial, while excessive or insufficient stiffness may elevate injury risk [18,19].

Tensiomyography (TMG) has emerged as a promising tool for evaluating post-exercise muscle stiffness without inducing additional fatigue [2]. A recent study by Rey et al. [2] investigated changes in tensiomyographic variables over a microcycle in professional male soccer players and examined their relationship with training load. The findings indicated an increase in muscle stiffness immediately following training, with players able to restore mechanical function before the subsequent session. This suggests that stiffness monitoring could be a valuable tool for managing neuromuscular load and mitigating injury risk. However, to date, no studies have explored these relationships in female soccer players.

In light of the limitations associated with existing methods, newer tools such as the MyotonPRO (Myoton AS, Tallinn, Estonia) have been developed. The MyotonPRO is more portable and user-friendly than TMG, and its reliability in measuring muscle mechanical properties, including stiffness and elasticity, has been well-documented [20,21]. Research indicates that myometric parameters exhibit strong correlations with both muscle force production and activation [22]. These findings suggest that myotonometry may assist practitioners in optimizing performance and rehabilitation programs while minimizing injury risk [22,23].

Therefore, this study aimed to assess changes in myometric parameters over a training microcycle in semi-professional female soccer players. In addition, the training load variables during the mycrocicle were analyzed, exploring their potential relationship to the changes in myotonometric parameters. We hypothesize that post-training stiffness changes will occur and that these changes will correlate with the magnitude of the training load.

## 2. Materials and Methods

### 2.1. Design

A repeated measures design was used to evaluate changes in muscle contractile properties (muscle stiffness, elasticity, and tone) assessed with the MyotonPRO over the course of a training microcycle during the competitive phase of the season in semi-professional female soccer players. The study lasted six days, including four training sessions classified according to the number of days since the last competitive match. The sessions were labeled MD1 (1 day after the match), MD3 (3 days after the match), MD4 (4 days after the match), and MD5 (5 days after the match). The variables assessed with the MyotonPRO device were measured in the biceps femoris (BF) and rectus femoris (RF) muscles, both before and immediately after each training session. Training load was quantified by session duration, RPE, and GPS. All training sessions and tests were performed at the club’s training facilities.

### 2.2. Participants

A total of 19 semi-professional soccer players (age: 23.9 ± 3.9 years; body mass: 60.6 ± 6.9 kg; height: 164.5 ± 6.7 cm) who belonged to the same female soccer team competing in the Spanish third division participated in this study. A priori power analysis was performed using G*Power software (version 3.1.9.2, Universität Kiel, Düsseldorf, Germany) to calculate sample size, assuming a Type I error of 0.05 and a Type II error rate of 0.20 (80% statistical power). The analysis determined that a minimum of 16 participants would be required to detect medium effects (partial eta squared; η_p_^2^ = 0.06). All participants had a minimum of two years and a maximum of twelve years of semi-professional soccer experience. Typical training for the players consisted of 4–5 full team workouts for a total training load of approximately 8–9 h per week and 35–50 min of effective time per session on the field plus two days of strength training in the gym with a mean duration of 40 min. The team also regularly competed in one official match per week. Since the physical load and specific training of the goalkeepers differed from the field players, they were not included in this study. Only players who completed all training sessions during the entire microcycle were considered for inclusion.

### 2.3. MyotonPro Measurements

The MyotonPRO (Myoton AS, Tallinn, Estonia) is a portable device that percutaneously applies a fast-release mechanical impulse to the muscle, resulting in muscle oscillations. The oscillations are then measured by an accelerometer and several parameters are calculated simultaneously. Myotometric measurements obtained with this system have demonstrated good validity, optimal relative and absolute reliability, and a high sensitivity to changes [24,25,26,27]. In addition, the intra- and inter-rater reliability of myotonometer measurements was found to be good to excellent (intraclass correlation coefficient > 0.75) in several studies [22,28]. The parameters calculated by MyotonPRO and used in this study are as follows:

- Dynamic stiffness (N/m): resistance to a contraction or an external force that deforms its initial shape [29].
- Tone (frequency of oscillation) (Hz): (intrinsic tension at the cellular level) of a muscle in its passive state. Describes the state of tension of a muscle in its contractile state [29].
- Logarithmic decrement (relative arbitrary unit): This metric characterizes the elasticity of tissue by measuring the dissipation of mechanical energy. Specifically, it assesses a tissue’s ability to recover its original shape after being deformed, either from contraction or the removal of an external force. A higher logarithmic decrement indicates lower elasticity [22,29].

The assessment was performed on the RF and BF of the dominant leg. The RF plays a crucial role during soccer’s sprinting, horizontal deceleration, cutting, jumping, and kicking actions [30,31,32]. Meanwhile, the BF has been measured due to its relevance in high-intensity actions and sprints across various motion trajectories [33,34]. Measurements were taken under static and relaxed conditions with the subject in supine and prone positions to measure the RF and BF, respectively. The anatomical location of the measurement points and the positions of the participants were standardized for all subjects and determined in accordance with previous studies [20,35,36]. With the subject in the supine position, the knee joint was fixed at an angle of 120° (180° corresponds to full knee extension). The measured limb was placed on a foam roller to keep the knee angle fixed. The measurement point for the rectus femoris was set at 33% of the total distance between the line formed between the top of the patella and the iliac spine from the distal part of the muscle, while the measurement point for the BF was set at 50% of the distance between the femoral condyle and the ischial tuberosity [23]. The location of the measurement point was marked with a semi-permanent marker pen to ensure the reliability of subsequent measurements. The measurement and subsequent marking of the measurement points was performed by a physiotherapist with more than 5 years’ experience. The standard 3 mm diameter probe was placed perpendicular to the skin surface, just above the muscle, and light pressure was applied with constant force until the light on the device turned green. A short (15 m·s^−^^1^), low force (0.4 N) mechanical impulse was applied, producing a constant compression of 0.18 N in the superficial subcutaneous tissue. This compression was then transmitted to the underlying muscle, and the subsequent damped oscillation of the muscle was recorded using an accelerometer [23,29]. The mean value of five consecutive measurements at each anatomical site was used for analysis [37]. If the coefficient of variation exceeded 3%, the measurement was repeated. All measurements were made by the same evaluator.

### 2.4. Training Load Quantification

To quantify the external training load during each session, each player wore a GPS (YOOMEDOO sports tracker v3; Yoomedoo SL, Palma de Mallorca, Spain) attached to the upper back using adjustable harnesses. The device has an accelerometer and gyroscope with a data rate of up to 250 Hz, and the sampling rate for the geolocation system was 10 Hz. The data were analyzed using the system-specific software (YOOMEDOO Software 3.0; yoomedoo SL, Palma de Mallorca, Spain). Similarly to previous studies carried out in both men’s and women’s soccer, the following variables were calculated during each training session: TD, average distance (TD covered divided by the effective training duration, expressed in m·min^−^^1^), HSRD, number of sprints, number of accelerations (>2 m·s^−^^2^) and number of decelerations (<−2 m·s^−^^2^) [2,38,39,40]. Both HRSD and sprint were individualized for each player according to their peak velocity measured during a 40 m sprint test [41]. To assess internal load and exercise intensity, players were asked to report their RPE using Foster’s 0–10 scale [42]. Players were shown the scale 10 min after each training session and asked, “How hard was your training?” All players were familiar with this scale as part of the normal monitoring of training sessions.

### 2.5. Statistical Analysis

A one-way repeated measures analysis of variance (ANOVA) was used to test for differences in each training load variable across the four training sessions, as well as to assess differences in the myotonometry percentage change variables. A two-way (session [MD1 vs. MD3 vs. MD4 vs. MD5] × time [pre-training vs. post-training]) repeated-measures ANOVA was used to analyze the changes in myotonometric parameters during the microcycle. In the case of a significant effect, Bonferroni-adjusted post hoc tests were used to identify differences. In addition, Cohen’s *d* effect sizes were calculated for all comparisons. Cohen’s *d* were classified as trivial (*d* < 0.2), small (0.2 ≤ *d* < 0.5), moderate (0.5 ≤ *d* < 0.8), and large (≥0.8) [43]. The relationship between training load variables during the microcycle and changes in myotonometric parameters was analyzed using Pearson’s correlation coefficient (*r*). Descriptive data are presented as mean with standard deviation (SD). Statistical significance was set at *p* < 0.05. All statistical tests were performed using JASP 0.14.1 statistical software (University of Amsterdam, Amsterdam, The Netherlands).

## 3. Results

### 3.1. Differences in Training Load

Table 1 shows the training load for each session during the testing period. The repeated measures ANOVA revealed a significant effect (*p* < 0.001) of microcycle day on RPE, TD, average distance, HSRD, number of sprints, and number of accelerations and decelerations. Bonferroni adjusted post hoc tests showed a lower RPE for MD1 compared to MD3 (*p* = 0.017; *d* = 1.27) and MD4 (*p* = 0.030; *d* = 1.27), and for MD5 compared to MD3 (*p* < 0.001; *d* = 1.49) and MD4 (*p* < 0.001; *d* = 1.49). For TD covered, significant differences were observed in MD1 compared to MD3 (*p* < 0.001; *d* = 2.00) and MD4 (*p* < 0.001; *d* = 2.35), and for MD5 compared to MD3 (*p* < 0.001; *d* = 2.78) and MD4 (*p* < 0.001; *d* = 3.13). For the average distance, a smaller distance per minute was observed in MD1 compared to MD3 (*p* < 0.001; *d* = 1.71) and MD4 (*p* < 0.001; *d* = 1.50), and a larger distance in MD3 and MD4 compared to MD5 (*p* < 0.001; *d* = 1.39 and 1.18). Regarding the HSRD, lower values were observed in MD1 compared to MD3 (*p* < 0.001; *d* = 1.26) and MD4 (*p* < 0.001; *d* = 2.40), as well as in MD3 compared to MD4 (*p* < 0.001; *d* = 1.14), whereas higher distances were observed in MD3 and MD4 compared to MD5 (*p* < 0.001; *d* = 1.00 and 2.14). Regarding the number of sprints, lower values were observed in MD1 (*p* < 0.001; *d* = 3.75), MD3 (*p* < 0.001; *d* = 3.47), and MD5 (*p* < 0.001; *d* = 3.92) with respect to MD4. A lower number of accelerations were performed in MD1 compared to MD3 (*p* = 0.024; *d* = 0.72) and MD4 (*p* < 0.001; *d* = 1.74), and in MD3 compared to MD4 (*p* < 0.001; *d* = 1.02). A higher number of accelerations were performed in MD3 (*p* = 0.033; *d* = 0.54) and MD4 (*p* < 0.001; *d* = 1.56) compared to MD5. Regarding decelerations, a lower number was realized in MD1 with respect to MD3 (*p* = 0.002; *d* = 1.15) and MD4 (*p* = 0.001; *d* = 1.10), as well as in MD5 compared to MD3 (*p* = 0.001; *d* = 0.88) and MD4 (*p* < 0.001; *d* = 0.83).

### 3.2. Differences in Myotonometric Parameters

**Figure 1:**
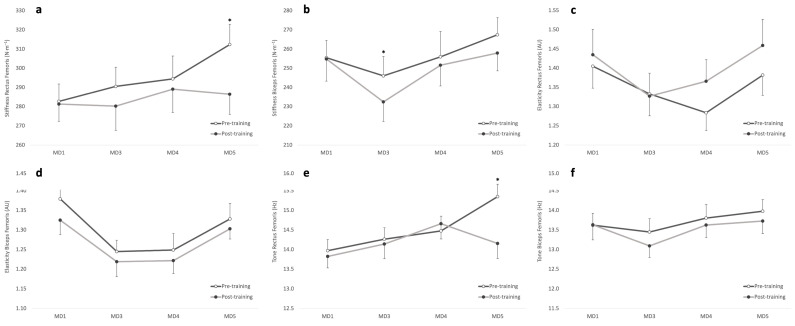
Differences in myotonometric parameters during a microcycle. * Significant differences between pre-training and post-training values. (**a**) Stiffness of rectus femoris; (**b**) stiffness of biceps femoris; (**c**) elasticity of rectus femoris; (**d**) elasticity of biceps femoris; (**e**) tone of rectus femoris; (**f**) tone of biceps femoris.

Figure 1 illustrates the differences in myotonometric parameters during a microcycle. For RF stiffness, a significant main effect was found for microcycle day (*p* = 0.015) and moment (*p* = 0.004) but not for the interaction day × moment (*p* = 0.080). Pre-training stiffness was significantly lower on MD1 compared to MD5 (*p* = 0.003; *d* = 0.65). Moreover, significant changes in RF stiffness values were observed in MD5 from pre- to post-training (*p* = 0.009; *d* = 0.57). In terms of BF stiffness, significant effects were observed for microcycle day (*p* = 0.002) and moment (*p* = 0.033) but not for the day × moment (*p* = 0.403) interaction. Post-training BF stiffness was significantly lower on MD3 compared to both MD1 (*p* = 0.047; *d* = 0.50) and MD5 (*p* = 0.017; *d* = 0.57). Significant pre- to post-training changes were also observed in MD3 (*p* = 0.048; *d* = 0.31).

Regarding RF elasticity, repeated measures ANOVA showed a significant effect for day (*p* = 0.006) but not for moment (*p* = 0.085) or the day × moment interaction (*p* = 0.594). For BF elasticity, a significant main effect was obtained for day (*p* < 0.001) and moment (*p* = 0.021) but not for the interaction day × moment (*p* = 0.838). Pre-training BF elasticity was higher on MD1 compared to MD3 (*p* = 0.002; *d* = 0.92) and MD4 (*p* = 0.004; *d* = 0.89), while post-training BF elasticity was also higher on MD1 than on MD3 (*p* = 0.042; *d* = 0.73).

For RF tone, significant effects were evidenced for microcycle day (*p* < 0.001), moment (*p* = 0.012), and the day × moment interaction (*p* = 0.007). With respect to BF, a significant effect was found for day (*p* = 0.015) but not for the moment (*p* = 0.101) or the day × moment interaction (*p* = 0.640). RF tone was significantly higher on MD5 pre-training compared to both MD1 (*p* < 0.001; *d* = 0.93) and MD3 (*p* = 0.007; *d* = 0.73). A significant pre- to post-training change was also observed on MD5 (*p* = 0.001; *d* = 0.81).

Finally, Table 2 shows the differences observed for the percentage change in stiffness, elasticity, and tone of the RF and BF. Repeated measures ANOVA revealed a significant effect (*p* = 0.012) of microcycle day on RF tone, with a greater percentage change at MD4 compared to MD5 (*p* = 0.011; *d* = 0.78). Moreover, there were significative correlations between changes in RF tone and training RPE (*p* = 0.025; *r* = 0.258) and TD (*p* = 0.006; *r* = 0.312).

## 4. Discussion

The main findings of this study are that: (a) significant differences were observed in the training load variables across different training sessions, with MD4 showing the highest values of HSRD, number of sprints and number of accelerations; MD3 showing the highest values of RPE; and MD5 showing the lowest values for TD covered; (b) significant differences were observed from pre- to post-training in RF and BF stiffness in MD5 and MD3, respectively, and RF tone in MD5; (c) significant differences were observed in myotonometric variables across training sessions during the microcycle; (d) significant differences were observed in the percentage change in muscle tone between training sessions during the microcycle.

The training load in this study was similar to those performed by various elite and highly trained women’s soccer teams, showing a similar trend in the microcycle structure characterized by an increase in daily training loads during the initial training sessions and a decrease in training load as the competition day approaches [11]. TD, number of sprints, and RPE are among the values referenced by most authors who have analyzed samples with similar characteristics [11]. The most notable difference observed regarding these studies was the reduction in the number of accelerations compared to the training load of a first-division Spanish team and the decrease in HSRD covered compared to a first-division team from the Australian league [44,45]. In this study, the highest absolute loads were observed in MD3 and MD4 (midweek days of the microcycle), while the lightest load was in MD5 (the last training session before the match), reflecting an attempt to expedite full recovery and fine-tuning of the players before the next match.

Furthermore, the quadriceps play an important role during ball striking, sprinting, and jumping, while the hamstrings control running activity and stabilize the knee during turns, changes in direction, and tackles [46]. Due to this, the quadriceps and hamstrings are the muscles most frequently injured during pre-season and competition season, respectively [46,47,48]. Previous research has shown that weekly training loads can alter muscle stiffness and that elevated stiffness levels prior to a training session are a discriminating factor in injury incidence [2,18,19]. However, it is still unclear how these variables influence stiffness in both male and female professional soccer players.

Muscle stiffness is defined as the amount of tension residing in the muscle–tendon unit and is calculated as the ratio between maximum force and the change in length of the muscle–tendon unit (force/deformation ratio) [19]. Our results showed that soccer training load caused a decrease in muscle stiffness in both the RF and BF, with significant results in MD5 (−8.18%) for RF and in MD3 (−5.32%) for BF. This may seem counterintuitive since it contradicts the initial hypothesis. Additionally, it contrasts with findings from other studies such as Rey et al. [2], which observed a significant increase in stiffness after all training sessions, except for MD5. The authors explain that the lack of stiffness increase in MD5 was due to reduced training loads. This leads us to believe that stiffness is modified based on the magnitude and type of training load and is dependent on the type of muscle contraction, as also supported by Rey et al. [2], who observed significant associations between post-session changes in RF Dm and training duration, high-speed distance, and average distance [2,49,50]. Therefore, in the present study, the training load may have been insufficient to produce increases in muscle stiffness. On the other hand, it could have been excessive in the previous weeks, leading to an accumulation of fatigue and being related to the behavior of stiffness in endurance sports, where changes in muscle contractile properties are less pronounced [51,52].

Moreover, it is important to note that similar previous studies have been conducted on male athletes, and the stiffness may behave differently in women. Therefore, the comparison of the obtained results loses validity, as potential neuromuscular differences should be considered [2,27,53]. According to Paravlic et al. [54], aside from their study, there are no other TMG studies on female soccer players. Therefore, it is necessary for future studies to examine the differences in neuromuscular profiles between male and female soccer players.

Based on the previously discussed findings, it is recommended, whenever possible, to regularly monitor athletes’ fatigue and recovery status through different methods, one of which is measuring stiffness with the MyotonPRO device and creating a personal profile for each player to observe how their muscle contractile properties vary and fluctuate. This would allow for better adjustment of the training load [50].

Moreover, elasticity, measured through the logarithmic decrement (D), characterizes tissue elasticity; that is, the ability of a tissue to recover its initial shape after contraction or the removal of an external deforming force, in this case, from muscle tissue [29]. Additionally, it describes the conditions of blood supply to the muscle during effort and the capacity to increase movement speed. A decrease in elasticity leads to faster muscle fatigue, and movement speed is limited if the muscle is less elastic [55]. Since muscle elasticity is inversely proportional to the D value, the lower the indicated value, the more elastic the muscle [56]. Therefore, RF elasticity decreased every day, particularly on MD4 (−7.09%), whereas BF elasticity increased post-training every day, especially on MD1 (−3.94%). If we consider the relationship between elasticity and stiffness, we find that it is dependent on the type of training and the primary muscle contraction type. In a study aimed at analyzing the effects of core balance training on muscle tone and balance ability in adults, a negative correlation was found between the two in males, meaning that when one increases, the other decreases. However, such correlation was not demonstrated in the female sample [56]. Conversely, a study comparing two types of muscle training (T1—explosive strength and plyometrics, T2—maximum strength and isometric exercises) using myometry to estimate muscle tone found that T1 increased elasticity, stiffness, and blood flow, so in this case, there would be no negative correlation. However, T2 increased stiffness but not elasticity [55].

Considering our results, we can observe that RF stiffness decreased every day, as did RF elasticity, though not coinciding when significant differences were present. However, BF elasticity increased every day, contrary to its stiffness. This leads us to hypothesize that the correlation between stiffness and elasticity is not only dependent on the type of training and contraction but also on the movement pattern and musculature. Nevertheless, it is challenging to contextualize the results of this study, as there is not enough literature discussing the subject, and in the limited existing studies, the samples and training programs are very different. Therefore, the behavior of elasticity and stiffness, both separately and together, remains unknown.

Muscle tone, measured through oscillation frequency (Hz), refers to the intrinsic tension at the cellular level of a muscle in its passive state. In other words, it describes the tension state of a muscle in a contractile state [29]. Additionally, it provides information about muscle recovery after exertion and muscle weakness [55]. The results of muscle tone evaluation through myotonometry can be an indicator of fatigue when correlated with certain enzymes, providing information about muscle metabolic processes and recovery [57]. In the study conducted by Rusu et al. [55] mentioned earlier, muscle tone increased with T1 training and decreased with T2 training, which the authors related to the greater accumulated fatigue in T2. In our case, muscle tone decreased every day except for MD4, being significant on MD5 (−7.54%) for RF. This may be related to the accumulated fatigue during the week of work, being higher on the last day. This may also be related to the decrease in stiffness, as explained earlier, since we believe it may have decreased post-training due to fatigue. Nevertheless, as we determined with elasticity, it is very complex to contextualize the results of muscle tone behavior due to the lack of literature on the subject.

With regard to the limitations of the study, it is important to highlight the lack of a control group, which would have allowed us to understand the players’ responses to the Myoton measurements without the influence of training load. In addition, it is important to test other muscle groups, such as the calf, in order to see more clearly whether the contractile properties change differently according to the muscles involved. Furthermore, if more than one microcycle could be measured, we would better understand the role of fatigue and training load on contractile properties. This also points to the importance of measuring the effects of competition on contractile properties to determine if the fatigue from the week of training alters the responses, making them subacute, or if they are simply acute responses to the load. Finally, the use of a biochemical variable such as creatine kinase (CK) would provide a better reference as an indirect marker of muscle damage, allowing better control of fatigue and recovery between sessions [50].

## 5. Conclusions

This study showed significant differences in training load variables between the different sessions, with MD4 showing the highest values for HSRD, number of sprints, and accelerations, while MD3 showed the highest values for RPE. In addition, significant pre- to post-training changes in stiffness were observed for RF in MD5 and BF in MD3, as well as for RF tone in MD5. Myotonometric variables also differed significantly between training sessions during the microcycle, highlighting the relationship between training load and these variables. Consequently, strength and conditioning coaches could monitor these variables as an indicator of players’ neuromuscular status, allowing them to adjust training across microcycles to prevent excessive fatigue and reduce the risk of non-contact injuries. However, further research is necessary to better understand how training load impacts these variables and to explore the role of gender.

## Figures and Tables

**Table 1:** Training load during the testing period.

Variable	MD1	MD3	MD4	MD5	*p*-Value	*Post Hoc*
Effective training time (min)	45	43	48	31		
RPE	3.7 ± 1.4	5.1 ± 1.1	5.1 ± 1.1	3.4 ± 1.1	<0.001	MD1, MD5 < MD3, MD4
TD (m)	3149.8 ± 938.9	4981.3 ± 752.2	5295.1 ± 928.1	2435.1 ± 917.0	<0.001	MD1, MD5 < MD3, MD4
Average distance (m·min^−1^)	69.9 ± 23.1	115.8 ± 17.5	110.3 ± 19.3	78.5 ± 29.5	<0.001	MD1, MD5 < MD3, MD4
HSRD (m)	82.6 ± 65.2	194.3 ± 100.7	294.7 ± 123.8	106.0 ± 39.0	<0.001	MD1, MD5 < MD3 < MD4
Sprints (n)	2.1 ± 3.7	4.9 ± 11.1	41.8 ± 17.7	0.2 ± 0.5	<0.001	MD1, MD3, MD5 < MD4
Accelerations (n)	16.4 ± 12.5	25.4 ± 14.7	38.4 ± 13.9	18.6 ± 8.4	<0.001	MD1, MD5 < MD3 < MD4
Decelerations (n)	10.6 ± 7.1	20.8 ± 11.6	20.4 ± 9.4	13.1 ± 6.6	<0.001	MD1, MD5 < MD3, MD4

RPE: Rate of perceived exertion; TD: total distance; HSRD: high-speed running distance.

**Table 2:** Percentage change in intra-session myotometric variables.

Variable		MD1	MD3	MD4	MD5	*p*-Value	*Post Hoc*
Stiffness	RF	−0.29 ± 6.34	−3.33 ± 13.34	−1.54 ± 10.89	−8.18 ± 7.54	0.107	
	BF	−0.61 ± 9.47	5.32 ± 9.47	−0.46 ± 11.77	−3.44 ± 6.93	0.327	
Elasticity	RF	3.82 ± 21.39	0.13 ± 11.89	7.09 ± 15.91	5.98 ± 15.17	0.604	
	BF	−3.94 ± 7.58	−1.95 ± 9.89	1.55 ± 8.25	−1.01 ± 9.49	0.778	
Tone	RF	−1.01 ± 3.95	−0.64 ± 9.89	1.50 ± 8.19	−7.54 ± 8.34	0.012	MD4 > MD5
	BF	0.01 ± 6.31	−2.22 ± 8.19	−1.05 ± 6.15	−1.75 ± 4.94	0.736	

RF: Rectus femoris; BF: Biceps femoris.

## Data Availability

The data presented in this study are available on request from the corresponding author. The data are not publicly available due to restrictions of the subjects’ agreement.
